# Human-Inspired Force-Motion Imitation Learning with Dynamic Response for Adaptive Robotic Manipulation

**DOI:** 10.3390/biomimetics10120825

**Published:** 2025-12-09

**Authors:** Yuchuang Tong, Haotian Liu, Tianbo Yang, Zhengtao Zhang

**Affiliations:** 1 CAS Engineering Laboratory for Intelligent Industrial Vision, Institute of Automation, Chinese Academy of Sciences, Beijing 100190, China; yuchuang.tong@ia.ac.cn (Y.T.); liuhaotian2021@ia.ac.cn (H.L.); yangtianbo2022@ia.ac.cn (T.Y.); 2 Beijing Zhongke Huiling Robot Technology Co., Ltd., Beijing 100192, China

**Keywords:** bioinspired robotics, imitation learning, force–motion skill acquisition, adaptive control

## Abstract

Recent advances in bioinspired robotics highlight the growing demand for dexterous, adaptive control strategies that allow robots to interact naturally, safely, and efficiently with dynamic, contact-rich environments. Yet, achieving robust adaptability and reflex-like responsiveness to unpredictable disturbances remains a fundamental challenge. This paper presents a bioinspired imitation learning framework that models human adaptive dynamics to jointly acquire and generalize motion and force skills, enabling compliant and resilient robot behavior. The proposed framework integrates hybrid force–motion learning with dynamic response mechanisms, achieving broad skill generalization without reliance on external sensing modalities. A momentum-based force observer is combined with dynamic movement primitives (DMPs) to enable accurate force estimation and smooth motion coordination, while a broad learning system (BLS) refines the DMP forcing function through style modulation, feature augmentation, and adaptive weight tuning. In addition, an adaptive radial basis function neural network (RBFNN) controller dynamically adjusts control parameters to ensure precise, low-latency skill reproduction, and safe physical interaction. Simulations and real-world experiments confirm that the proposed framework achieves human-like adaptability, robustness, and scalability, attaining a competitive learning time of 5.56 s and a rapid generation time of 0.036 s, thereby demonstrating its efficiency and practicality for real-time applications and offering a lightweight yet powerful solution for bioinspired intelligent control in complex and unstructured environments.

## 1. Introduction

Recent advances in robotic manipulators have enabled transformative applications across manufacturing, healthcare, and service industries. Nevertheless, achieving human-like dexterity and generalizable interaction in complex, unstructured environments remains a persistent challenge [1,2,3,4].

Imitation learning based on dynamic movement primitives (DMPs) offers an efficient and data-driven method to acquire task-specific motion skills [5,6,7,8]. However, unlike humans who can flexibly adapt motion and force responses to external disturbances, conventional DMP-based frameworks exhibit limited adaptability, revealing the need for more generalizable skill learning mechanisms [9,10,11]. To improve adaptability and obstacle avoidance, DMPs have been extended with potential fields, bioinspired acceleration terms, and adaptive feedback control, combined with online planning to facilitate cooperative manipulation [12,13,14,15,16]. Imitation learning has further enhanced skill refinement and transferability [17,18,19]. Nonetheless, current methods face notable limitations: obstacle avoidance DMPs often fail to generalize across humanoid tasks, stylistic DMPs show restricted adaptability to new contexts, and imitation learning rarely accounts for stylistic or adaptive variability [15,20,21,22]. Despite increasing applications of DMPs in cooperative control, studies addressing stylistically adaptive imitation learning that supports robust task execution and broad generalization remain scarce [23,24,25].

Human motor skills inherently couple motion trajectories with muscle stiffness and contact forces—factors critical for transferring dexterous skills to robots [20]. DMPs have therefore been extended to represent motion and stiffness profiles, enhancing the learning of compliant behavior [26]. However, while robotic motion can be readily tracked via position sensors, direct measurement of contact forces is often constrained by sparse sensor deployment [27,28]. Alternative methods, such as stiffness estimation or EMG-based inference, are prone to noise and modeling errors. Momentum-based force observers provide an effective alternative by estimating contact forces accurately without requiring joint acceleration measurements, maintaining robustness under dynamic uncertainties [29].

Recent developments in advanced control have further contributed to adaptive and robust robotic behavior. Techniques such as sliding-mode control, adaptive neural network (NN) control, and robust control have demonstrated strong potential for handling nonlinear and uncertain dynamics [30,31,32,33]. Neural and fuzzy systems have been widely adopted for adaptive control to compensate for unmodeled dynamics, with fuzzy logic improving NN adaptability under system uncertainties [34]. Adaptive fuzzy neural network (FNN) control has shown effectiveness in managing interaction and uncertainty [35,36,37]. However, the presence of large payloads or time-varying dynamics can induce biases that degrade the performance of radial basis function neural networks (RBFNNs), reducing tracking accuracy in position and velocity control [38,39,40].

To address these challenges, this paper presents a human-inspired imitation learning framework that unifies force–motion learning and dynamic response mechanisms, enabling robots to robustly acquire and generalize manipulation skills in dynamic, contact-rich environments. Compared to conventional teaching-by-demonstration methods and previous approaches that separately model motion and force or rely on external sensors such as force/EMG [7,9,10,19,41], our bioinspired imitation learning framework simultaneously acquires motion and force skills, adaptively generalizes across tasks and environments without additional sensors, and dynamically responds to environmental changes, enabling robust, compliant, and human-like manipulation. In contrast to existing SoTA methods [11,26,28,31,33] that depend on data-intensive learning or sensor-heavy hybrid control, our method delivers comparable adaptability and precision while requiring substantially lower sensing and computational resources, thereby preserving the interpretability, modularity, and stability inherent in structured control paradigms. At its core, the framework integrates a momentum-based force observer with dynamic movement primitives (DMPs) for accurate force–motion coupling, augmented by a broad learning system (BLS) that refines the DMP forcing function through style modulation, feature augmentation, and adaptive weight tuning. An adaptive RBFNN controller further modulates control parameters in real time, ensuring precise and low-latency skill reproduction under unanticipated disturbances. This unified, lightweight, and sensor-free architecture significantly enhances robustness, adaptability, and scalability, providing a practical and generalizable solution for adaptive manipulation and human–robot interaction in uncertain, unstructured environments. The main contributions are summarized as follows:

1. We propose a human-inspired imitation learning framework that jointly acquires and generalizes force–motion skills, enabling robots to perform robust, adaptive, and compliant manipulation in dynamic, contact-rich environments, thereby addressing key challenges in practical robotic applications.
2. A momentum-based force observer integrated with DMPs and BLS is developed to enhance force–motion coupling, refine skill trajectories through style modulation and feature augmentation, and achieve accurate, low-latency skill reproduction.
3. An adaptive RBFNN controller is introduced to dynamically tune control parameters in response to unforeseen disturbances, improving system robustness, scalability, and safe physical interaction in unstructured environments.

This paper is organized as follows: Section 2 introduces the DMP-based skill modeling. Section 3 details imitation learning and the adaptive RBFNN controller. Section 4 overviews the framework. Section 5 presents simulation and experimental results. Section 6 concludes this paper.

## 2. Hybrid Force-Motion Skill Learning

### 2.1. Sensorless Momentum-Driven Force Observers

To acquire force-related skills from human demonstration maneuvers, a momentum-based force observer is employed to estimate the human contact forces without relying on external force sensors. The robot dynamics are described by

(1) $$\begin{array}{c} D\left(q\right)\ddot{q}+S\left(q,\dot{q}\right)\dot{q}+G\left(q\right)+\tau_{e}=\tau_{c} \end{array}$$

where $D\left(q\right)\in R^{n\times n}$, $S\left(q,\dot{q}\right)\dot{q}\in R^{n}$, and $G\left(q\right)\in R^{n}$ represent the inertia matrix, Coriolis and centripetal vector, and gravity vector, respectively. Here, *n* denotes the number of degrees of freedom, and *q*, $\dot{q}$, and $\ddot{q}$ denote the joint position, velocity, and acceleration. The terms $\tau_{c}$ and $\tau_{e}$ represent the control torque and the external torque (e.g., human-induced), respectively.

The generalized momentum of the manipulator is defined as $p=D\left(q\right)\dot{q}$. Taking its time derivative yields

(2) $$\dot{p}=\dot{D}\left(q\right)\dot{q}+D\left(q\right)\ddot{q}=\dot{D}\left(q\right)\dot{q}-S\left(q,\dot{q}\right)\dot{q}-G\left(q\right)+\tau_{c}-\tau_{e},$$

which simplifies to

(3) $$\dot{p}=S^{T}\left(q,\dot{q}\right)\dot{q}-G\left(q\right)+\tau_{c}-\tau_{e},$$

using the identity $\dot{D}\left(q\right)-2S\left(q,\dot{q}\right)=-S^{T}\left(q,\dot{q}\right)$.

To analyze how each component of $\dot{p}$ is affected by its corresponding torque, we expand the expression element-wise as

(4) $$\begin{array}{c} {\dot{p}}_{i}=-\frac{1}{2}{\dot{q}}^{T}\frac{\partial D(q)}{\partial q_{i}}\dot{q}-G_{i}\left(q\right)+\tau_{c,i}-\tau_{e,i},i=1,\ldots ,n. \end{array}$$

Based on this, the momentum observer is constructed as

(5) $$\begin{array}{c} \begin{array}{cc} \dot{\hat{p}} & =\tau_{c}-\hat{\Lambda }\left(q,\dot{q}\right)+\eta \\ \dot{\eta } & =K_{e}\left(\dot{p}-\hat{\hat{p}}\right) \end{array} \end{array}$$

where $\hat{p}$ is the estimated momentum and η is the observer output. The compensating term $\Lambda (q,\dot{q})$ simplifies dynamics calculation and is defined as

(6) $$\begin{array}{c} \begin{array}{cc} \Lambda (q,\dot{q}) & :=S\left(q,\dot{q}\right)\dot{q}-\dot{D}\left(q\right)\dot{q}+G\left(q\right)\\ \; & =-S^{T}\left(q,\dot{q}\right)\dot{q}+G\left(q\right) \end{array} \end{array}$$

Here, $K_{e}=\mathrm{diag}\left(k_{e,1},\ldots ,k_{e,n}\right)$ is a positive-definite gain matrix. Integrating the observer dynamics yields

(7) $$\begin{array}{c} \begin{array}{cc} \eta & =K_{e}\left(p\left(t\right)-\int_{0}^{t}\dot{\hat{p}}\left(t\right)dt-p\left(0\right)\right)\\ \; & =K_{e}\left(p\left(t\right)-\int_{0}^{t}\left(\tau_{c}-\hat{\Lambda }\left(q,\dot{q}\right)+\eta \right)dt-p\left(0\right)\right) \end{array} \end{array}$$

Assuming ideal model knowledge (i.e., $\hat{D}\left(q\right)=D\left(q\right)$, $\hat{\Lambda }\left(q,\dot{q}\right)=\Lambda \left(q,\dot{q}\right)$), the error dynamics simplify to

(8) $$\begin{array}{c} \dot{\eta }=K_{e}\left(\tau_{e}-\eta \right) \end{array}$$

Applying the Laplace transform to this first-order system yields

(9) $$\begin{array}{c} \eta_{i}=\frac{k_{e,i}}{t+k_{e,i}}\tau_{e,i}=\frac{1}{1+T_{e,i}s}\tau_{e,i},\;i=1,\ldots ,n \end{array}$$

Thus, a larger gain $k_{e,i}$ results in a faster transient response (i.e., smaller time constant $T_{e,i}$). In the limit as $K_{e}\to \infty$, the observer output η asymptotically models the actual external torque $\tau_{e}$.

Hence, the momentum observer effectively functions as a virtual torque sensor, which allows for estimations of external Cartesian forces $F_{e}$ through the robot’s Jacobian matrix. To reduce noise in the estimated force signal, a Kalman filter [42] is employed, with its output modeled as

(10) $$\begin{array}{c} F_{e}\left(t\right)=H\left(t\right)\hat{\lambda }\left(t\right) \end{array}$$

where $\hat{\lambda }\left(t\right)$ denotes the estimated Wiener filter coefficients, and H(t) is the corresponding virtual regressor.

**Remark** **1.** *The generalized momentum-based force observer [42] estimates external torques without inverting the inertia matrix or coupling DOFs, enabling robust, sensorless contact force estimation that reduces noise and enhances data quality and learning efficiency during human demonstrations.*

### 2.2. Skill Encoding with Dynamic Movement Primitives

To encode motion skills observed from human demonstrations, the DMP formulation is employed as follows

(11) $$\begin{array}{cc} \; & \tau_{d}\dot{v}=\alpha_{x}\left({𝒳}_{g}-𝒳\right)-\beta_{x}v+\alpha_{f}\left[f\left(s\right)-\left({𝒳}_{g}-{𝒳}_{0}\right)s\right]\\ \; & \tau_{d}\dot{𝒳}=v \end{array}$$

where $𝒳,v,\dot{v}$ denote the position, velocity, and acceleration in Cartesian space, respectively. Subscripts _0_ and _g_ indicate the initial and goal positions. The constants $\alpha_{x},\beta_{x},\alpha_{f}\in R$ are positive scalars, and $\tau_{d}>0$ is a temporal scaling factor.

This system (11) can be interpreted as a spring-damper mechanism modulated by a virtual force term $\alpha_{f}\left[f\left(s\right)-\left({𝒳}_{g}-{𝒳}_{0}\right)s\right]$, where $\left({𝒳}_{g}-{𝒳}_{0}\right)$ acts as a spatial scaling term. The phase variable *s* evolves according to a canonical system

(12) $$\begin{array}{c} \tau_{d}\dot{s}=-\varphi s,\;\varphi >0,\;s_{0}=1 \end{array}$$

where φ denotes the decay rate.

The nonlinear forcing term f(s) is represented as a weighted sum of normalized Gaussian basis functions

(13) $$\begin{array}{c} f\left(s\right)=\sum_{i=1}^{N_{\psi }}\omega_{i}\psi_{i}\left(s\right)s \end{array}$$

with

(14) $$\begin{array}{c} \psi_{j}\left(s\right)=\frac{\exp\left[-{\left(s-b_{j}\right)}^{2}/\left(2c_{j}\right)\right]}{\sum_{j=1}^{N_{\psi }}\exp\left[-{\left(s-b_{j}\right)}^{2}/\left(2c_{j}\right)\right]} \end{array}$$

where $\omega_{j}$ are the corresponding weights, and $b_{j}$ and $c_{j}$ denote the mean and variance of the *j*-th basis function, respectively. $N_{\psi }$ is the total number of basis functions. As *s* monotonically decays to zero from its initial value $s_{0}>0$, both f(s) and the virtual forcing term converge to zero, resulting in $𝒳\to {𝒳}_{g}$.

Assuming the demonstration trajectory is generated according to Model (11), the weights $\omega_{j}$ can be learned using linear regression. The expected forcing term is derived as

(15) $$\begin{array}{cc} f^{*}\left(s\right)= & \frac{\tau_{d}\ddot{𝒳}\left(\nabla s\right)+\beta_{x}\dot{𝒳}\left(\nabla s\right)}{\alpha_{x}}\\ \; & \;-\left({𝒳}_{g}-𝒳\left(\nabla s\right)\right)+\left({𝒳}_{g}-{𝒳}_{0}\right)s \end{array}$$

where 𝒳(·) denotes the demonstration trajectory, and ∇s is the inverse of $s\left(t\right)=s_{0}\exp\left(-\varphi t/\tau_{d}\right)$. The parameter vector $\Omega =[\omega_{1},\omega_{2},\ldots \omega_{N_{\psi }}]$ can be efficiently identified using the least-squares method based on the data obtained from Equation (14).

To capture force-related skills in the demonstrations, we similarly encode the evolution of external force $F_{e}$ as a trajectory using a DMP-based model

(16) $$\begin{array}{c} \begin{array}{cc} \tau_{d}{\dot{F}}_{v} & =\alpha_{x}\left(F_{g}-F\right)-\beta_{x}F_{v}+\alpha_{f}\left[f\left(s\right)-\left(F_{g}-F_{0}\right)s\right]\\ \tau_{d}\dot{F} & =F_{v} \end{array} \end{array}$$

where *F* and $F_{v}$ denote the force profile and its rate of change. While the parameters $\alpha_{x}$, $\beta_{x}$ and $\alpha_{f}$ in this model are specific to the force dynamics, the temporal scaling factor $\tau_{d}$ and the phase variable *s* are shared with Model (11) to maintain temporal alignment.

**Remark** **2.** *The DMP-based models (11) and (14) use spring-damper systems driven by virtual forces for motion and force, balancing rigidity and flexibility akin to human muscle stiffness modulation. Tuning $x_{g}$ and $F_{g}$ enables precise motion and force goals, while $\tau_{d}$ controls task duration, forming a unified, generalizable framework for encoding human motor behavior.*

## 3. Skill Generalization & Reproduction

### 3.1. BLS-Based Forcing Function Modulation

The BLS [43] enhances training efficiency by mapping inputs into feature nodes and augmenting them with enhancement nodes. Integrated with DMPs, this method improves adaptability to disturbances in dynamic environments. Given input I and output $H\in R^{N_{f}\times M}$, the output is modeled as

(17) $$\begin{array}{c} H=\left[{ℱ}^{n_{z}}|{ℰ}^{n_{e}}\right]𝒲=𝒵𝒲, \end{array}$$

where feature nodes $ℱi=\phi_{i}\left(IWfi+\gamma_{fi}\right)$ and enhancement nodes $ℰj=\xi_{j}\left({ℱ}^{n_{z}}Wej+\gamma_{ej}\right)$ capture nonlinear transformations. Output weights 𝒲 are efficiently computed via pseudo-inverse.

Adding new enhancement nodes ${ℰ}^{n\_e+1}$ allows incremental weight updates:

(18) $$\begin{array}{c} {𝒲}^{N_{f}+1}=\left[\begin{array}{c} {𝒲}^{N_{f}}-{𝒵}^{+}{ℰ}^{n_{e}+1}\kappa^{T}H\\ \kappa^{T}H \end{array}\right], \end{array}$$

with $\kappa^{T}$ defined based on residuals ϱ and projections ρ. This structure enables fast, adaptive learning with improved feature representation.

To enhance the expressiveness of the original forcing term f(s), we introduce an extended formulation $f^{\mathrm{new}}\left(s\right)$ within the BLS framework. A subset of Gaussian basis functions from the original set is retained, while additional features $\chi_{j}\left(s\right)=\zeta_{j}\left(\Phi \left(s\right)W_{j}+\gamma_{j}\right)$ are incorporated. Here, $\zeta_{j}$ is a nonlinear activation function, and $W_{j},\gamma_{j}$ are randomly initialized weights and biases, designed to enrich the representational capacity of the state vector Ψ(s). The augmented state components $\mu_{k}$ are normalized as

(19) $$\begin{array}{c} \mu_{k}=\frac{l_{k}\left(s\right)\psi_{k}\left(s\right)+\left(1-l_{k}\left(s\right)\right)\chi_{k-n_{z}}\left(s\right)}{\sum_{i=1}^{n_{z}}\psi_{i}\left(s\right)+\sum_{j=1}^{n_{e}}\chi_{j}\left(s\right)}s, \end{array}$$

where $l_{k}\left(s\right)=1$ if $k\leq n_{z}$, and 0 otherwise. This ensures that $\sum_{k=1}^{n_{z}+n_{e}}\mu_{k}=s$.

Defining $\Pi \sum_{i=1}^{n_{z}}\psi_{i}\left(s\right)=\sum_{i=1}^{n_{z}}\psi_{i}\left(s\right)+\sum_{j=1}^{n_{e}}\chi_{j}\left(s\right)$, the expression simplifies to

(20) $$\begin{array}{c} \mu_{k}=\frac{l_{k}\left(s\right)\psi_{k}\left(s\right)}{\Pi }+\frac{\left(\Pi -1\right)\left(1-l_{k}\left(s\right)\right)\chi_{k-n_{z}}\left(s\right)s}{\Pi \sum_{j=1}^{n_{e}}\chi_{j}\left(s\right)}. \end{array}$$

The enhanced state vector Ξ(s) is constructed as

(21) $$\begin{array}{c} \Xi \left(s\right)={\left[\mu_{1},\mu_{2},\ldots ,\mu_{n_{z}+n_{e}}\right]}^{T}={\left[\frac{\Psi (s)}{\Pi }|\left(1-\frac{1}{\Pi }\right)\Upsilon \left(s\right)\right]}^{T}, \end{array}$$

with Υ(s) denoting normalized enhanced components $\varsigma_{j}\left(s\right)=\frac{\chi_{j}\left(s\right)s}{\sum_{j}\chi_{j}\left(s\right)}$. The new forcing term is then defined by

(22) $$\begin{array}{c} f^{\mathrm{new}}\left(s\right)={\left(\Omega_{n_{z}+n_{e}}^{\mathrm{new}}\right)}^{T}\Xi \left(s\right), \end{array}$$

where $\Omega^{\mathrm{new}}={\left[\Omega |\Omega^{𝒰}\right]}^{T}$ combines weights from the original and enhanced components. This yields

(23) $$\begin{array}{cc} f^{\mathrm{new}}\left(s\right) & =\frac{1}{\Pi }f\left(s\right)+\left(1-\frac{1}{\Pi }\right)f^{𝒰}\left(s\right)\\ \; & =f\left(s\right)+\left(\frac{1}{\Pi }-1\right)\left(f\left(s\right)-f^{𝒰}\left(s\right)\right). \end{array}$$

Assuming the additional compensation targets mid-segment of the trajectory, the system dynamics are modified as

(24) $$\begin{array}{cc} \; & \tau_{d}\dot{v}=\alpha_{x}\left(x_{g}-x\right)-\beta_{x}v+\alpha_{f}\left[f^{\mathrm{new}}\left(s\right)-\left(x_{g}-x_{0}\right)s\right],\\ \; & \tau_{d}\dot{x}=v. \end{array}$$

This structure supports incremental learning by allowing trajectory adaptation via the residual term

(25) $$\begin{array}{cc} \Delta 𝒞(s) & =f^{\mathrm{new}}\left(s\right)-f\left(s\right)=\left(\frac{1}{\Pi }-1\right)\left(f\left(s\right)-f^{𝒰}\left(s\right)\right)\\ \; & ={\left(\Omega^{\mathrm{new}}\right)}^{T}\Xi^{E}\left(s\right), \end{array}$$

where $\Xi^{E}\left(s\right)={\left[\left(1/\Pi -1\right)\Psi \left(s\right)|\left(1-1/\Pi \right)\Gamma \left(s\right)\right]}^{T}$ represents the extended state.

For each DoF, the desired enhanced term is computed by

(26) $$\begin{array}{c} f_{j}^{\mathrm{new}}{\left(s\right)}^{𝒟}=\frac{\tau_{d}{\ddot{x}}_{j}^{\mathrm{new}}+\beta_{x}{\dot{x}}_{j}^{\mathrm{new}}}{\alpha_{x}}-\left(x_{g}-x_{j}^{\mathrm{new}}\right)+\left(x_{g}-x_{0}\right)s. \end{array}$$

The corresponding weights are obtained by minimizing the discrepancy

(27) $$\begin{array}{c} \min\sum_{j=1}^{n_{z}}{∥f_{j}^{\mathrm{new}}{\left(s\right)}^{𝒟}-f_{j}\left(s\right)∥}^{2}. \end{array}$$

The trajectory compensation term becomes

(28) $$\begin{array}{cc} & \Delta {𝒞}_{j}^{\mathrm{new}}{\left(s\right)}^{𝒟}=f_{j}^{\mathrm{new}}{\left(s\right)}^{𝒟}-f_{j}{\left(s\right)}^{𝒟}\\ \; & \;=\frac{\tau_{d}\left({\ddot{x}}_{j}^{\mathrm{new}}-{\ddot{x}}_{j}\right)+\beta_{x}\left({\dot{x}}_{j}^{\mathrm{new}}-{\dot{x}}_{j}\right)}{\alpha_{x}}+\left(x_{j}^{\mathrm{new}}-x_{j}\right), \end{array}$$

and the final optimization objective is

(29) $$\begin{array}{c} \min\sum_{j=1}^{m}{∥\Delta {𝒞}_{j}^{\mathrm{new}}{\left(s\right)}^{𝒟}-\Delta {𝒞}_{j}\left(s\right)∥}^{2}. \end{array}$$

**Remark** **3.** *By introducing enriched features $\chi_{j}\left(s\right)$, the proposed method expands the state and parameter space, implicitly adapting the normalization factor* Π*. Guided by BLS theory, this extension enables accurate approximation of complex nonlinear mappings and facilitates rapid, flexible trajectory refinement under dynamic conditions.*

While the previous formulation supports adaptation via a single compensation term, it remains limited in representing diverse motion variations. To address this, we propose a generalized formulation of the compensation terms $\Delta {𝒞}^{k}\left(s\right),\;k=1,\ldots ,𝒦$, each capturing a distinct motion modulation based on a shared baseline trajectory f(s)

(30) $$\Delta {𝒞}^{k}\left(s\right)={\left(\Omega_{n_{z}+n_{e}}^{\mathrm{new}}\right)}^{k}\;\Xi^{E}\left(s\right),$$

where ${\left(\Omega_{n_{z}+n_{e}}^{\mathrm{new}}\right)}^{k}$ denotes a unique set of weights associated with the *k*-th modulation mode, and $\Xi^{E}\left(s\right)$ is the common extended state-vector defined in (21).

To model these variations more compactly, we introduce a set of style coefficients ${𝒮}_{k}\in R$, such that

(31) $$\Delta {𝒞}^{k}\left(s\right)={𝒮}_{k}\;\Omega_{n_{z}+n_{e}}^{\mathrm{new}}\;\Xi^{E}\left(s\right)={𝒮}_{k}\;\Delta {𝒞}^{𝒮}\left(s\right),$$

with the style modulation matrix defined as

(32) $$S=[\;{𝒮}_{1},\;{𝒮}_{2},\;\ldots ,\;{𝒮}_{𝒦}\;],$$

which encodes inter-demonstration variability and enables adaptive modulation of the DMP forcing term. Each coefficient ${𝒮}_{k}$ is empirically determined from demonstration data by minimizing the deviation between the baseline DMP forcing term and the observed trajectory via least-squares regression. A BLS is then employed to refine the estimates of ${𝒮}_{k}$, thereby capturing stylistic variations across multiple demonstrations and ensuring accurate trajectory reproduction and generalization.

To obtain these coefficients, we reformulate the imitation learning model by embedding a style-attractor landscape, replacing the conventional forcing function [44]. Given the target modulation set $\Delta ℱ{\left(s\right)}^{𝒟}=\left[\Delta {𝒞}_{1}{\left(s\right)}^{𝒟},\ldots ,\Delta {𝒞}_{𝒦}{\left(s\right)}^{𝒟}\right]$, we apply singular value decomposition

(33) $$\begin{array}{c} \Delta ℱ{\left(s\right)}^{𝒟}=U\Sigma V^{T}\approx S\Delta {ℱ}^{S}{\left(s\right)}^{𝒟}, \end{array}$$

where $\Delta {ℱ}^{S}{\left(s\right)}^{𝒟}=\left[\Delta {𝒞}_{1}^{S}{\left(s\right)}^{𝒟},\ldots ,\Delta {𝒞}_{𝒦}^{S}{\left(s\right)}^{𝒟}\right]$ denotes the style-invariant basis.

The optimal weights ${\left(\Omega_{n_{z}+n_{e}}^{\mathrm{new}}\right)}^{k}$ are then learned by minimizing the reconstruction loss

(34) $$\begin{array}{c} \min\sum_{k=1}^{𝒦}∥\Delta {ℱ}_{k}^{S}{\left(s\right)}^{𝒟}-\Delta {ℱ}_{k}^{S}\left(s\right)∥. \end{array}$$

**Remark** **4.** *Integrating BLS with DMP in an imitation learning framework yields a unified representation for diverse motions. The normalized shared state vector $\Xi^{E}\left(s\right)$ enables scalable weight expansion and compact style modulation, supporting flexible, context-aware trajectory adaptation.*

### 3.2. Adaptive Control with RBFNN

This paper utilizes an RBFNN-based controller to accurately track both motion and force profiles corresponding to learned behaviors, encompassing both routine and feedback-driven executions, thereby enabling skill replication. Initially, the trajectories obtained from the baseline behavior (11) and those generalized from interaction-induced variations (24) are assigned as the desired joint positions $Q_{d}\in R^{n}$ and velocities ${\dot{Q}}_{d}\in R^{n}$. The tracking errors are defined as

(35) $$\begin{array}{c} E=Q_{d}-q,\dot{E}={\dot{Q}}_{d}-\dot{q}. \end{array}$$

where *n* denotes the number of DOFs in joint space.

To improve both the stability and tracking performance, a virtual control law is designed as

(36) $$\begin{array}{c} 𝒱={\dot{Q}}_{d}+{𝒫}_{1}Q \end{array}$$

where ${𝒫}_{1}=diag\left\{p_{q,1},\ldots ,p_{q,n}\right\}$ is a diagonal and positive definite gain matrix. The vector Q is defined based on L’Hospital’s rule as

(37) $$\begin{array}{cc} Q & ={\left[\sigma_{1},\ldots ,\sigma_{n}\right]}^{T}\\ \; & ={\left[\frac{\iota_{1}^{2}}{2\pi E_{1}}\sin\left(\frac{\pi E_{1}^{2}}{\iota_{1}^{2}}\right),\ldots ,\frac{\iota_{n}^{2}}{2\pi E_{n}}\sin\left(\frac{\pi E_{n}^{2}}{\iota_{n}^{2}}\right)\right]}^{T} \end{array}$$

where ι is a constant vector.

The control input is constructed as

(38) $$\begin{array}{c} \tau_{c}={𝒫}_{2}𝒱+W^{T}\;\cdot \;\Phi \left(𝒜\right) \end{array}$$

where ${𝒫}_{2}$ is another diagonal gain matrix, W denotes the adaptive weight matrix, $𝒜={\left[{𝒜}_{1},{𝒜}_{2},\ldots {𝒜}_{q}\right]}^{T}\in \Theta_{𝒜}\subset R^{q}$ is the neural input, and $\Phi =[\phi_{1},\phi_{2},\ldots ,\phi_{n}]$ represents the basis function set. The generalized inner product ⋄ is defined as

$$W^{T}⋄\Phi \left(𝒜\right):={\left[W_{1}^{T}\phi_{1}\left(𝒜\right),W_{2}^{T}\phi_{2}\left(𝒜\right),\ldots ,W_{n}^{T}\phi_{n}\left(𝒜\right)\right]}^{T}.$$

This neural approximation term can model any bounded continuous function to arbitrary precision given sufficient nodes, according to

(39) $$\begin{array}{c} ℵ\left(𝒜\right)=W_{i}^{T}\phi_{i}\left(𝒜\right)+\epsilon_{i}\left(𝒜\right),\forall 𝒜\in \Theta_{𝒜} \end{array}$$

where i=0,l,g denote networks with no bias, local bias, and global bias respectively; $\epsilon_{i}\left(𝒜\right)$ is the approximation error.

The Gaussian basis function used is

(40) $$\begin{array}{c} \phi_{j}\left(𝒜\right)=\exp\left[-\frac{{\left(𝒜-h_{j}\right)}^{T}\left(𝒜-h_{j}\right)}{r^{2}}\right],\;j=1,2,\ldots ,n, \end{array}$$

where $h_{j}$ is the center vector of the *j*-th hidden node, and *r* is the width parameter.

The optimal weights are defined by minimizing the worst-case approximation error:

(41) $$\begin{array}{c} W^{*}:=\arg\min_{W}\left\{\underset{𝒜\in \Theta_{𝒜}}{\sup}\left|ℵ\left(𝒜\right)-W^{T}\Phi \left(𝒜\right)\right|\right\}. \end{array}$$

where ℵ(𝒜) is the approximate model of the target dynamics.

The neural model approximates the dynamics (1) as

(42) $$\begin{array}{c} \begin{array}{c} W^{*T}⋄\Phi \left(𝒜\right)+\epsilon \left(𝒜\right)=D\left(q\right)\ddot{q}+S\left(q,\dot{q}\right)\dot{q}+G\left(q\right)+\tau_{e} \end{array} \end{array}$$

where the input $𝒜=\left[q^{T},{\dot{Q}}_{d}^{T},{\ddot{Q}}_{d}^{T}\right]$ reduces the dimensionality to 3n, thereby decreasing the hidden node complexity from $m^{4n}$ to $m^{3n}$. Fine-tuned control gains are used to mitigate residual approximation errors.

The online learning law adopts a gradient descent with a discontinuous δ-modification:

(43) $$\begin{array}{c} {\dot{W}}_{k}=L_{k}\left(\phi_{k}\left(𝒜\right)r_{k}-\delta_{k}W_{k}\right) \end{array}$$

where $L_{k}$ is the learning rate, and the switching gain $\delta_{k}$ is defined as

(44) $$\begin{array}{c} \delta_{k}=\left\{\begin{array}{cc} 0 & \;\mathrm{if}\;∥W_{k}∥<{\bar{W}}_{k}\\ {\bar{\delta }}_{k} & \;\mathrm{if}\;∥W_{k}∥⩾{\bar{W}}_{k} \end{array}\right. \end{array}$$

Since the true optimal weights $\left|W^{*}k\right|$ are unknown, $\bar{W}k$ must be empirically tuned. A value too small effectively reduces the algorithm to fixed damping, while a value too large risks weight drift and control instability in high-gain settings [45].

**Remark** **5.** *While δ-modification is commonly used to enhance the robustness of adaptive RBFNN controllers [35,45,46], its fixed damping effect can undermine function approximation. This drawback is mitigated via a discontinuous switching scheme, wherein $\delta_{k}$ is deactivated (i.e., set to zero) when the weight norm $\left|W_{k}\right|$ falls below a predefined threshold ${\bar{W}}_{k}$, allowing more accurate adaptation without sacrificing stability.*

## 4. Overview of the Framework

**Figure 1:**
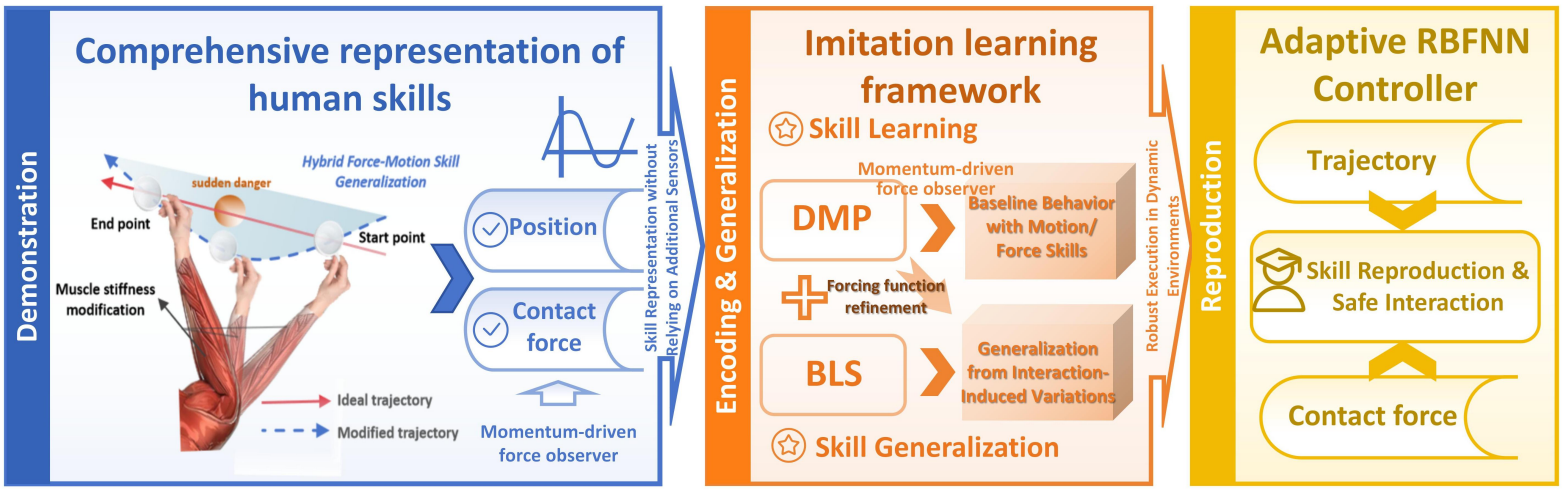
Block diagram of the incremental learning framework for enhancing robotic responsiveness through integrated hybrid force-motion learning and dynamic response mechanisms.

The proposed bioinspired imitation learning framework for motor and interaction skill acquisition consists of three stages (Figure 1), enabling robots to learn, generalize, and execute motion and force behaviors in dynamic, unstructured environments. The framework draws inspiration from human motor control principles, emphasizing adaptive force–motion coupling, style-based skill generalization, and reflex-like responsiveness.

In the demonstration and encoding phase, human demonstrations under unstructured contact conditions are captured using a momentum-based force observer, without relying on external force or electromyography sensors. Essential kinematic and dynamic data are encoded as Dynamic Movement Primitives (DMPs) by extracting parameters such as weight vectors ω, basis activations ψ(s), and nonlinear forcing terms f(s) via Equations (11) and (12), yielding a compact representation that closely approximates target trajectories (26). This stage enables human-like adaptive coordination between motion and force, allowing reflexive responses to environmental perturbations that extend beyond the capabilities of conventional TbD methods.

For skill generalization, the framework integrates a Broad Learning System (BLS) with DMPs, incrementally refining internal models through adaptive modulation terms $\Delta ℱ{\left(s\right)}^{𝒟}$ computed by Equations (26) and (28) and decomposed into stylistic components $\Delta {ℱ}_{k}^{S}{\left(s\right)}^{𝒟}$ via singular value decomposition. Using prior weights Ω and extended state vectors $\Xi^{E}\left(s\right)$, the adapted weights $\Omega_{n_{z}+n_{e}}^{\mathrm{new}}$ are optimized following Equations (19)–(22), (31), and (32), allowing context-sensitive modulation of motion and force. Style coefficients ${𝒮}_{k}$ and BLS integration enable reproduction of demonstration variability, capturing human-inspired trajectory nuances and ensuring robust generalization across tasks and environments. Compared to traditional TbD robots, this approach allows simultaneous learning of motion and force skills, supporting compliant, adaptive interaction in high-contact tasks and enabling skill generalization without requiring additional demonstrations or sensors.

Finally, an RBFNN controller dynamically adjusts parameters to track desired joint-space trajectories $Q_{d}\in R^{n}$ and velocities ${\dot{Q}}_{d}\in R^{n}$, reducing input dimensionality from 4n to 3n to enhance computational efficiency. This adaptive control layer preserves smooth, low-latency motion while maintaining stable force–motion coordination under unforeseen environmental changes, such as obstacle appearance or surface variations. Collectively, these bioinspired elements endow the robot with reflexive adaptability, sensor-free efficiency, human-like skill generalization, and robust responsiveness to dynamic perturbations, demonstrating clear advantages over standard teaching-by-demonstration approaches.

## 5. Simulation and Physical Experiments

This section presents simulations and physical experiments with the UR5 manipulator to demonstrate the framework’s effectiveness and practicality in diverse tasks.

### 5.1. Simulation Setup

Simulation experiments were conducted in Matlab and CoppeliaSim to validate the proposed framework using manually collected demonstrations. In the simulated task, the robot autonomously performs whiteboard drawing while avoiding obstacles, with contact forces estimated via a momentum-based observer. Hybrid force-position skill learning ensures precise trajectory tracking, while the incremental learning mechanism facilitates trajectory diversification and enhances generalization.

**Figure 2:**
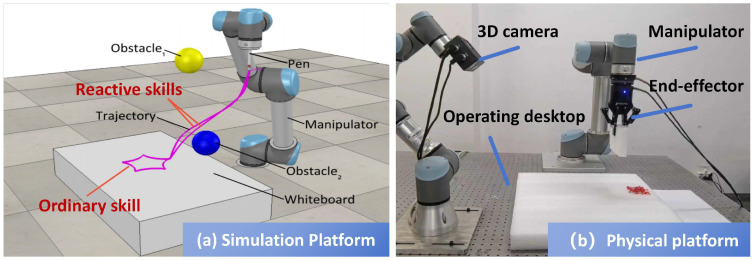
Simulation and physical platforms.

The physical experimental platform (Figure 2) consists of a UR5 manipulator, a 3D camera, and an operator console. The 3D camera serves solely as an environmental perception module for auxiliary tasks, including target localization, obstacle detection, and workspace boundary monitoring, ensuring safe and reliable task execution. It does not participate in the force–motion control loop or the skill generalization process. Obstacle configuration in simulation adheres to predefined workspace safety and reachability principles: (1) obstacles are placed within the manipulator’s reachable workspace without violating kinematic or joint constraints; (2) sufficient spacing between obstacles is maintained to preserve maneuverability; (3) obstacle positions are randomized across trials to evaluate the generalization capability of the learned skill; and (4) irregular or non-convex obstacles are approximated as convex polyhedra with spherical envelopes to enable computationally tractable collision checking. These setup rules ensure consistent, reproducible conditions for evaluating reactive and compliant motion generation under diverse and realistic scenarios.

The main experimental parameters are configured as follows: α=60, β=15, the number of basis functions is 200, and the initial number of enhancement nodes is 1. The initial and final joint states are defined as $q\left(0\right)={\left[0.657,\;-1.195,\;0.392,\;-0.768,\;-1.571,\;0\right]}^{T}\;\mathrm{rad}$ and $\dot{q}\left(0\right)={\left[0,\;0,\;0,\;0,\;0,\;0\right]}^{T}\;\mathrm{rad}/s$. Obstacles are modeled as spheres with a radius of 0.05m, located at (−0.45, −0.175, 0.6)m and (−0.425, −0.275, 0.275)m, respectively, with a minimum obstacle distance of $d_{\min}=0.06\;m$. The total task duration is set to t=30s.

### 5.2. Verification of the Momentum-Driven Force Observer

**Figure 3:**
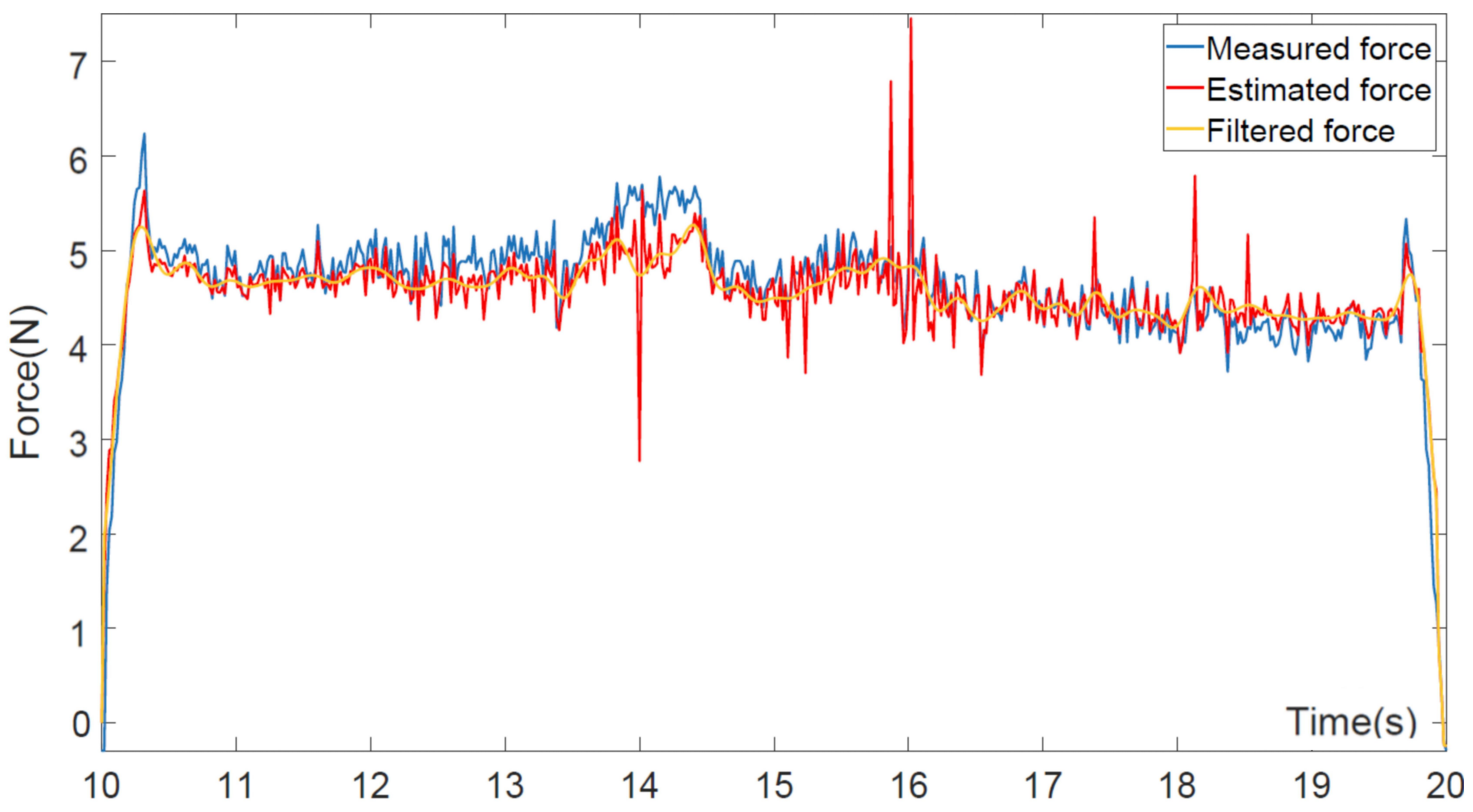
Estimated and measured contact forces for momentum-based force observers.

Figure 3 evaluates the proposed momentum-based force observer under conditions of intentionally applied constant external force during the drawing task. The comparison between sensor-measured force, estimated force, and Kalman-filtered output shows that, upon pen–whiteboard contact, the force rises sharply and then stabilizes. The sudden fluctuations observed in the estimated force primarily result from rapid transitions in contact states, including abrupt directional and positional adjustments of the end-effector. Around 14 s, a transient deviation between the filtered and measured forces occurs due to a short-lived, high-frequency contact disturbance. These transient peaks are effectively attenuated by the observer dynamics and the DMP-based motion smoothing mechanism. The momentum observer functions as a virtual torque sensor, estimating external Cartesian forces via the robot’s Jacobian matrix. Kalman filtering mitigates measurement noise and further enhances signal smoothness. Despite temporary peaks, the steady-state force estimation error remains on the order of $10^{-2}\;N$, without materially affecting task execution or the stability of adaptive control. This high estimation precision enables consistent reproduction of human-like force modulation, demonstrating the observer’s reliability and effectiveness in contact-rich robotic tasks.

### 5.3. Verification of the Incremental Learning Framework

**Figure 4:**
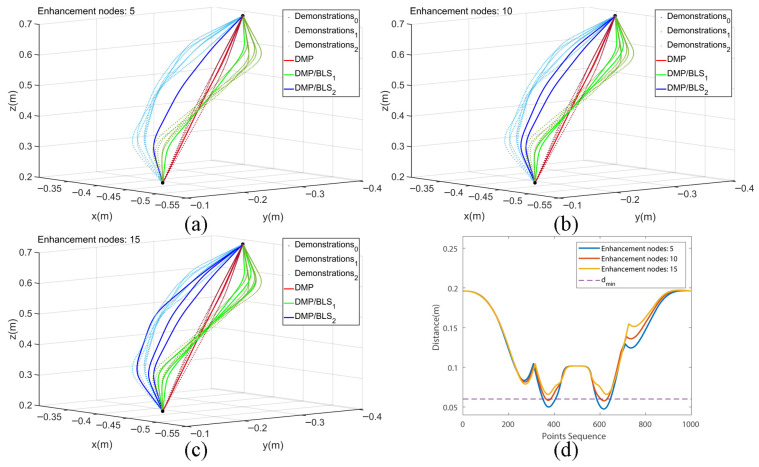
Trajectory learning process for reactive skills when the number of augmentation nodes increases. (**a**) performance of 5 enhancing nodes; (**b**) performance of 10 enhancing nodes; (**c**) performance of 15 enhancing nodes; (**d**) relationship between obstacle avoidance performance and the number of enhanced nodes.

Figure 4 illustrates the effectiveness of the proposed framework in acquiring reactive skills and achieving obstacle-aware motion adaptation. As shown in Figure 4a–c, the generated trajectories progressively converge toward smooth, collision-free motions as the number of augmentation nodes increases, demonstrating enhanced adaptability and refinement of the learned skill. Figure 4d further shows that, with an increasing number of augmentation nodes, the minimum distance between the manipulator and obstacles gradually exceeds the predefined safety threshold, indicating improved compliance and robustness in reactive behavior. This trend verifies that higher augmentation node density enhances the stability and safety margin of the learned skill. Collectively, these results substantiate the framework’s capability to generalize across multiple demonstrations, adapt to dynamic and contact-rich environments, and ensure safe and reliable physical interaction. The observed trajectory evolution further confirms that the integration of style modulation and adaptive learning enables robust, human-like reactive skill acquisition. Figure 4 therefore provides both qualitative and quantitative validation of the framework’s effectiveness in achieving safe, adaptive, and generalizable skill learning.

Furthermore, Table 1 quantitatively compares the proposed method with DMP [5], GMM-DMP [12], ProDMP [13], and CDPMM-DMP [16], demonstrating superior performance in trajectory similarity (i.e., the average distance of the generated trajectory to demo), learning time, and generation time. The main parameters of these methods are of the same size. As the number of enhanced nodes increases, our method achieves the trajectory similarity error of 0.020 m, indicating high fidelity in replicating demonstrated skills. Additionally, it maintains a competitive learning time of 5.56 s and a rapid generation time of 0.036 s, showcasing its efficiency and practicality for real-time applications. These results underscore the framework’s effectiveness in skill acquisition and generalization, outperforming existing methods across key performance metrics.

**Figure 5:**
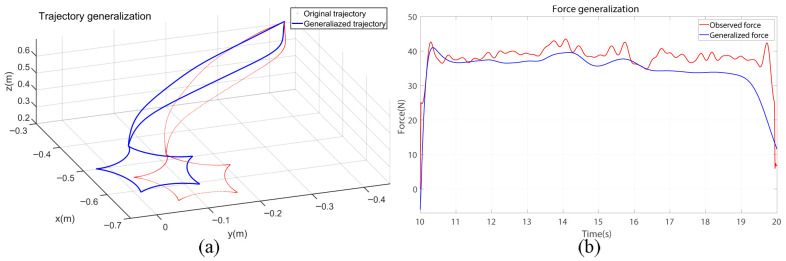
Analysis of the generalization ability of the incremental learning framework. (**a**) trajectory generation performance; (**b**) force generation performance.

An experiment altering the drawing position while keeping the original demonstration validated the framework’s generalization capability (Figure 5). Adjusted DMP trajectories enabled effective robot control using generalized trajectory and force data, demonstrating adaptability to spatial variations without major reconfiguration. This flexibility ensures precise, robust performance across diverse tasks and dynamic environments. By minimizing retraining or manual tuning, the framework enhances robotic efficiency and scalability. Overall, Figure 5 confirms the framework’s effectiveness in extending skill learning to new spatial contexts, supporting versatile and reliable robotic operation.

### 5.4. Verification of the Adaptive RBFNN Controller

**Figure 6:**
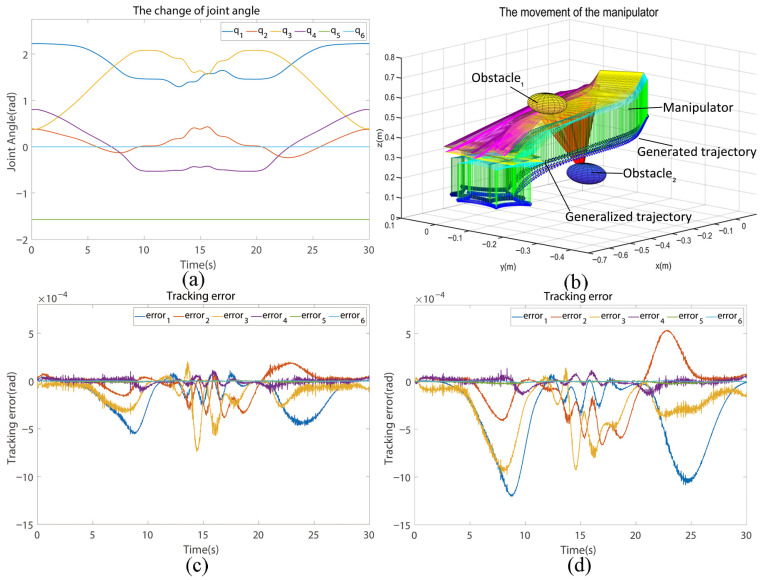
Tracking control results for skill reproduction using the adaptive RBFNN controller. (**a**) joint angle of the manipulator, (**b**) motion trajectory of the manipulator, (**c**) tracking error of the proposed adaptive RBFNN controller, and (**d**) tracking error of the conventional RBFNN controller.

Figure 6 validates this imitative learning framework’s precision and robustness in trajectory tracking. As shown in Figure 6a–c, the manipulator achieves smooth joint transitions and maintains tracking errors below $5\times 10^{-4}$ rad. The comparison in Figure 6b between learned and generalized trajectories confirms accurate path following and effective obstacle avoidance. Force-feedback-induced fluctuations in Figure 6c are well controlled, demonstrating stability in dynamic environments. Figure 6d further highlights the adaptive RBFNN controller’s superior accuracy and resilience compared to the conventional RBFNN, underscoring enhanced adaptability. Collectively, these results confirm the framework’s robustness and practical efficacy in precise, reliable execution of complex tasks under diverse conditions.

### 5.5. Implementation & Verification

**Figure 7:**
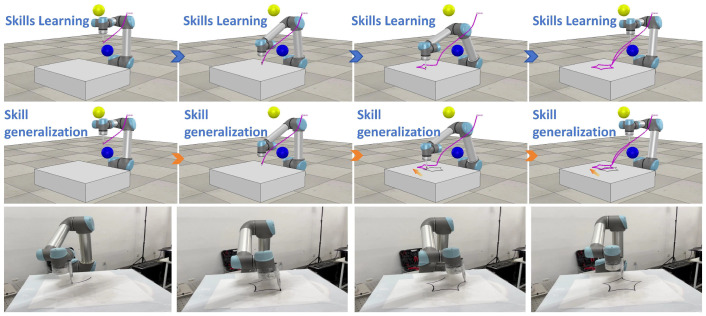
Simulation and experimental demonstrations. The 1st line: skill learning performance; the 2nd line: skill generalization performance; the 3rd line: physical experiment performance.

**Figure 8:**
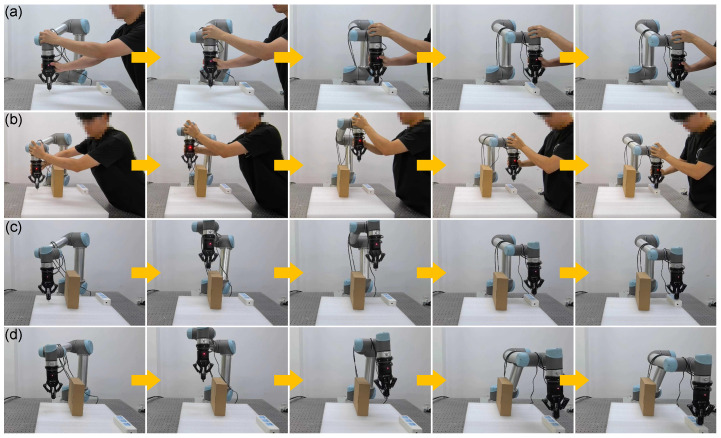
Physical experiments for skill learning and generalization in plug-in tasks. (**a**) data collection for obstacle-free case; (**b**) data collection for obstacle avoidance case; (**c**) skill learning performance; (**d**) skill generalization performance.

**Figure 9:**
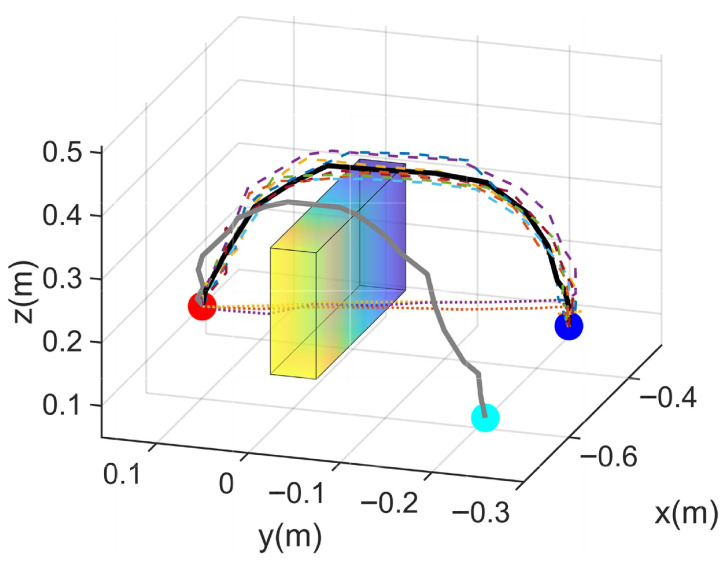
Trajectories of skill learning and generalization generation in physical experiments with plug-in tasks.

**Figure 10:**
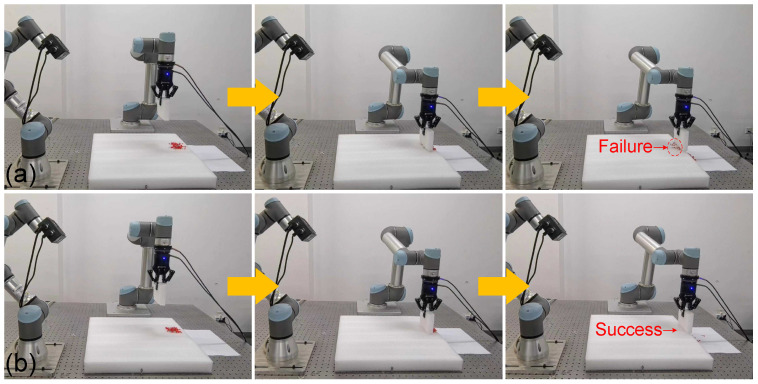
Comparison of skill learning in cleaning tasks through physical experiments. (**a**) Conventional position control; (**b**) the proposed method.

The results from both simulation and real-world experiments (Figure 6, Figure 7, Figure 8, Figure 9 and Figure 10) comprehensively validate the proposed imitation-learning framework in terms of skill generation, generalization, and robustness.

In simulation, Figure 6b and Figure 7 demonstrate that the learned trajectories successfully achieve collision-free performance across multiple randomly generated spherical obstacle configurations. The proposed augmentation mechanism enables trajectories to adaptively reshape in response to varying obstacle layouts, ensuring reliable obstacle-aware behavior under complex spatial constraints. The simulations further illustrate that reactive skill learning remains smooth and stable, allowing the manipulator to complete the specified end-effector drawing tasks despite the presence of multiple obstacles.

For physical validation, diverse manipulation tasks—including power-plugging and surface cleaning—were performed on the UR5 platform (Figure 8, Figure 9 and Figure 10). During skill acquisition, the robot accurately executes drawing and insertion tasks while maintaining safe distances from surrounding obstacles. When task locations or obstacle arrangements change, the system exhibits strong adaptability, generalizing learned motion and force profiles with minimal retraining. In the power-plugging task (Figure 8a–d), the framework generalizes human demonstrations to achieve collision-free insertion even when socket positions shift, aided by visual calibration from the 3D camera. The trajectories are adaptively generated via force–motion modulated Dynamic Movement Primitives (DMPs), and rather than representing a single globally optimal path, multiple feasible trajectories exist for the same task, each maintaining skill-consistent, compliant, and smooth force–motion execution. This demonstrates that the framework not only reproduces demonstrated skills but also flexibly responds to dynamic environmental changes, a capability often lacking in conventional teaching-by-demonstration robots. The robot consistently completes the insertion task while avoiding obstacles, and trajectory comparisons (Figure 9) confirm that the learned force–motion policy preserves stable, low-latency performance under varying conditions. In the cleaning task, interaction-force modulation (Figure 10b) ensures consistent surface contact and superior performance compared with conventional position control (Figure 10a), which often fails under force uncertainty. Collectively, these results indicate that the proposed framework effectively learns and reproduces force–motion skills, enabling precise, compliant, and adaptive interactive manipulation in tasks requiring both contact accuracy and dynamic environmental responsiveness.

Collectively, these findings substantiate that the proposed framework preserves stable force–motion coordination, smooth trajectory tracking, and compliant interaction in both single- and multi-obstacle environments. The framework demonstrates strong robustness against workspace perturbations and irregular obstacle geometries, verifying its capacity for safe, adaptive, and generalizable manipulation in complex and cluttered settings.

## 6. Conclusions

This paper presented a bioinspired imitation learning framework that unifies force–motion learning with dynamic response mechanisms, enabling robots to acquire and generalize dexterous manipulation skills in dynamic, contact-rich environments while operating with a lightweight, sensor-free architecture that substantially reduces sensing and computational requirements compared with existing SoTA methods. By integrating a momentum-based force observer with DMPs and a BLS, complemented by an adaptive RBFNN controller, the framework achieves accurate force estimation, low-latency skill reproduction, and robust, compliant performance without relying on external sensors. Simulation and real-world experiments demonstrate human-like adaptability and dexterity, safe physical interactions, and broad task generalization. These results underscore the framework’s potential as a practical, scalable solution for advanced robotic manipulation in unstructured and uncertain environments, providing a foundation for future extensions such as multi-robot cooperation, complex object handling, and vision-guided autonomy.

## Figures and Tables

**Table 1:** Quantitative comparisons among different methods.

Method	Similarity (m)	Learning Time (s)	Generation Time (s)
Ours	0.020	5.56	0.036
DMP [5]	0.043	22.45	0.045
GMM-DMP [12]	0.019	4.10	0.056
ProDMP [13]	0.031	1.72	0.033
CDPMM-DMP [16]	0.027	6.72	0.042

## Data Availability

Data are contained within the article.
